# Bio-Based Brewer’s Spent Grain Particleboards: Effect of Rosin Coating and Wood Veneer Reinforcement on Structure and Properties

**DOI:** 10.3390/polym18182303

**Published:** 2026-09-20

**Authors:** Lucia Rossi, Federico Rueda, Emiliano M. Ciannamea, Pablo M. Stefani

**Affiliations:** Instituto de Investigaciones en Ciencia y Tecnología de Materiales (INTEMA), Consejo Nacional de Investigaciones Científicas y Técnicas (CONICET), Universidad Nacional de Mar del Plata (UNMdP), Av. Colón 10850, Mar del Plata B7600FDQ, Argentina; luciarossig@fi.mdp.edu.ar (L.R.); federico.rueda@fi.mdp.edu.ar (F.R.); emiliano@fi.mdp.edu.ar (E.M.C.)

**Keywords:** brewer’s spent grain, biobased adhesive, wood, particleboard, mechanical properties, physical properties, X-ray microtomography, digital image correlation

## Abstract

Brewer’s spent grain (BSG) is an abundant agro-industrial by-product with potential for manufacturing sustainable particleboards, although its relatively low cellulose content limits the mechanical performance of the resulting boards. In this study, particleboards were produced from BSG using a soybean protein concentrate (SPC)-based adhesive at three adhesive contents. In addition, rosin surface coating and wood veneer reinforcement were investigated as strategies to improve the performance of the boards. Physical and mechanical properties were evaluated according to American and European standards, while X-ray micro-computed tomography was used to analyze the structure of the panels. Increasing the SPC content significantly improved all mechanical and physical properties. Rosin coating further reduced water absorption and moisture diffusivity while improving the modulus of rupture, modulus of elasticity, and internal bond strength. X-ray micro-CT analysis revealed local density increases associated with rosin penetration into the board structure. The greatest improvement was achieved with wood veneer reinforcement particleboards, satisfying the H1-ANSI A208.1 and P2-EN312 minimum flexural requirements. Moreover, Digital Image Correlation (DIC) revealed that wood veneer reinforcement significantly altered the deformation and failure mechanisms. These results demonstrate that simple surface reinforcement strategies enable the production of bio-based particleboards with significantly improved performance.

## 1. Introduction

The growing demand for sustainable alternatives to conventional wood-based materials has intensified research into the valorization of agricultural residues, particularly for the manufacture of engineered wood products, such as particleboards and fiberboards [1,2,3]. Several agroindustrial lignocellulosic residues, including rice husks, wheat straw, rapeseed straw, oil palm trunk, hemp, and sugarcane bagasse, have been successfully used in particleboard production due to their availability, low cost, and chemical composition [3,4,5,6,7,8,9,10,11].

Among these residues, brewer’s spent grain (BSG) has emerged as a promising candidate for particleboard production [9,11,12,13]. The brewing industry generates substantial quantities of BSG as its primary byproduct, creating significant disposal challenges. In Argentina alone, the craft beer sector produces approximately 20 million hectoliters annually, generating an estimated 400,000 tons of spent grain waste per year, based on the ratio that every 1 hectoliter of beer production yields 20 kg of BSG [14,15]. Although BSG has traditionally been utilized as livestock feed because of its high fiber and protein content, its high moisture content and susceptibility to microbial spoilage result in rapid deterioration and odor generation, creating significant logistical challenges, especially in urban areas with limited livestock production [16].

BSG exhibits a composition of 9.5% cellulose, 28.7% hemicellulose, 11.2% lignin and 17.3% protein [9]. In contrast, commercial woods used in particleboard manufacturing typically contain 40–45% cellulose, 25–30% hemicellulose, and 25–35% lignin [3,17]. BSG exhibits lower cellulose and lignin contents compared with wood, which may reduce its stiffness. Nevertheless, BSG appears to be a promising lignocellulosic substrate for bio-based adhesives, such as soy protein adhesives, due to its unique composition, which combines residual proteins with hydroxyl-containing polysaccharides. These components may contribute to interfacial adhesion by promoting intermolecular interactions and hydrogen bonding during hot pressing. Supporting this hypothesis, Zeng et al. (2023) demonstrated a synergistic effect between barley brewer’s grain proteins and soy protein isolate, showing that proteins derived from barley grain can actively participate in the development of high-performance bio-based adhesive systems [18]. Previous studies have demonstrated the feasibility of producing particleboards from agricultural residues using bio-based adhesives. In our prior work, we evaluated the processing parameters for manufacturing binderless particleboards from BSG [9]. These materials may be suitable for non-structural applications, such as ceiling panels, where the mechanical requirements are less demanding. Rice husk particleboards bonded with soy protein-based adhesives have achieved modulus of rupture (MOR) values of 12.4–17 MPa and modulus of elasticity (MOE) of 2.6–2.8 GPa [19], meeting the minimum requirements for commercial medium-density particleboards specified by ANSI A208.1 standard. Similarly, *Rhizophora* spp. particleboards using modified soy protein isolate exhibited MOR values ranging from 3.4–18.6 MPa and MOE 1.3–7.6 GPa [20]. Bagasse-based particleboards bonded with protein-based adhesives have also shown particularly impressive results, with MOR values reaching 1–6 MPa and MOE 0.4–0.8 GPa [21].

Conventional particleboard manufacturing still relies on synthetic adhesives, predominantly urea-formaldehyde and phenol-formaldehyde resins, which raise significant environmental and health concerns due to their toxic formaldehyde emissions. This has created an urgent need to develop more environmentally friendly binding alternatives [22]. Soy protein concentrate has attracted increasing attention as a bio-based adhesive due to its renewability, low toxicity, and reduced emissions of volatile organic compounds (VOCs) [23,24,25,26]. In Argentina, the abundant availability of soybeans further supports the use of this crop for adhesive production, offering a renewable and non-toxic alternative that aligns with circular economy principles and sustainable development goals [27,28,29]. For example, soy-based adhesives have already been used for engineering products as binders for conventional plywoods [30,31]. Moreover, the intrinsic protein content of BSG may synergistically interact with soy protein-based adhesives, potentially improving interfacial bonding between particles and reducing the overall adhesive requirement compared to traditional wood-based boards. In addition, processing parameters (pressure, temperature and time) have also been shown to significantly influence the performance of soy protein-based adhesive [22].

To further improve the physical and mechanical properties of particleboards, various treatments and additives, such as impregnation with oils and bio-based additives, have been explored [32,33,34]. The use of beeswax has shown effectiveness as a hydrophobic agent in medium-density fiberboards, while the use of a combination of pine rosin with aluminum salts has been shown to preserve mechanical properties and improve water resistance [35]. Rosin, a natural product mainly composed of a mixture of abietic acid and other resin acids, has also been reported as a protective waterproofing coating because it is an abundant, biodegradable, and low-cost resource [36,37]. Other approaches have focused on improving the mechanical performance of particleboards through the incorporation of natural fiber reinforcements. In particular, bidirectional jute fabric has been successfully incorporated into rice husk particleboards bonded with soybean protein concentrate (SPC), resulting in improved flexural and tensile properties [38].

Despite its potential as a sustainable lignocellulosic raw material, BSG presents inherent limitations for structural particleboard applications. Compared with wood, BSG contains substantially lower cellulose content and a higher proportion of hemicelluloses, resulting in particles with lower intrinsic stiffness and strength. Consequently, particleboards produced from BSG generally exhibit lower modulus of elasticity (MOE) and modulus of rupture (MOR), making it difficult to satisfy the mechanical requirements established for load-bearing or furniture applications [9,39]. One promising strategy to overcome these limitations is the incorporation of wood veneers as external reinforcement layers. In sandwich-like structures, the face layers are subjected to the highest tensile and compressive stresses during bending, while the core primarily resists shear stresses [40]. Therefore, placing high-stiffness wood veneers on the outer surfaces enables a more efficient distribution of stresses, significantly increasing the flexural stiffness and strength of the composite without substantially modifying its weight or the utilization of agricultural residues.

Overall, the use of BSG in particleboard production represents a valuable opportunity to reduce agricultural waste, decrease excessive wood consumption, and valorize agro-industrial residues within a circular economy approach, while promoting the use of bio-based adhesive systems as alternatives to synthetic adhesives. The combination of BSG, SPC-based adhesives, and suitable reinforcement or protective strategies offers a promising pathway toward the development of bio-based particleboard materials with reduced reliance on conventional wood and synthetic adhesives.

Our previous work demonstrated the feasibility of producing binderless particleboards using BSG as the unique material, and compared their performance with boards prepared with a phenolic adhesive [9]. However, the mechanical performance of the binderless boards remained limited, particularly for applications requiring higher flexural strength. The present study therefore addresses a different approach: the use of a soybean protein concentrate (SPC)-based adhesive to improve particle bonding, combined with surface strategies aimed at overcoming the specific limitations of BSG-based boards. In particular, rosin coating was investigated as a simple approach to improve moisture resistance, whereas wood veneer reinforcement was evaluated as a structural strategy to enhance flexural performance. The combined use of mechanical testing, X-ray micro-computed tomography and Digital Image Correlation further allows the effects of these strategies on board structure, deformation and failure behavior to be examined.

## 2. Materials and Methods

### 2.1. Materials

Brewer’s spent grains (BSG) obtained from Pilsner malt were provided by a local beer industry in Mar del Plata (Buenos Aires, Argentina). Soybean protein concentrate (SPC, Solcon S) was provided by Cordis S.A. (Villa Luzuriaga, Buenos Aires, Argentina) with a mean composition of 69% protein, 15% non-starch polysaccharides, 7% moisture, 5% ash, 3% fiber and 1% fat, with an average particle size that could pass through a 100-mesh sieve. Boric acid (H_3_BO_3_) was purchased from the Sigma Chemical Co. (St. Louis, MO, USA) (purity ≥ 99.5%, analytical grade reagent). Ethanol (EtOH, C_2_H_6_O, 96%, Porta, Córdoba, Argentina). Rosin (Colophony) resin (CL) was kindly supplied by Agroforestal Oberá SRL (Ituzaingó, Corrientes, Argentina). *Melia azedarach* wood veneer was obtained from sawmills in Misiones, Argentina.

### 2.2. BSG Conditioning

BSG was dried to a moisture content of approximately 5 wt% at 80 ± 2 °C in an air-circulating oven (Memmert, Lucé, France) and then stored in hermetically sealed plastic containers at room temperature until use. The chemical composition of the BSG consisted of 9.5% cellulose, 28.7% hemicellulose, 11.2% lignin, 17.3% protein, 3.3% ash, and 7.5% fat, with a particle size ranging from 500 to 6500 µm, as previously reported by Rossi et al. (2024) [9].

### 2.3. Conditioning and Characterization of Wood Veneer

The 0.64 ± 0.02 mm thick veneers were produced by flat slicing (knife slicing), avoiding the lathe checks typically associated with rotary-peeled veneers. The veneers were conditioned in a climate-controlled chamber at 20 ± 2 °C and 65 ± 2% relative humidity (RH) until equilibrium moisture content was reached. The moisture content and density of the conditioned veneer were determined according to ASTM D4442-07 and ASTM D2395-02, respectively. The tensile strength (TS) and tensile modulus of elasticity (TMOE) were determined in directions parallel (0°) and perpendicular (90°) to the wood fiber orientation, following a procedure adapted from Krüger et al. (2026) [41]. Rectangular specimens measuring 200 mm in length and 30 mm in width were used. The specimens were clamped over 30 mm at each end, resulting in a free gauge length of 140 mm. To prevent premature failure and localized crushing in the gripping region, both ends of the specimens were reinforced with end tabs prior to testing. Tensile tests were carried out using an INSTRON-EMIC 2350 universal testing machine (São José dos Pinhais, Brazil) equipped with a clip-on extensometer (Epsilon, Jackson, MI, USA). Tests were performed under displacement control at a crosshead speed of 1 mm/min.

### 2.4. Soybean Protein Concentrate-Based Adhesive Preparation

Following the procedure described by Ciannamea et al. (2012) [23], SPC was modified by dispersing it in a 3% (*w*/*v*) boric acid solution (BAS) at an SPC:BAS ratio of 1:10 under stirring (500 rpm) at room temperature for 2 h. The resulting dispersion was then frozen and freeze-dried. Two adhesive formulations were subsequently prepared by redispersing the freeze-dried SPC-BAS in water. An SPC-BAS-to-water ratio of 1:10 (BSPC) was employed for mixing with dried BSG, whereas a ratio of 1:7 (BSPC7) was used to impregnate the wood veneer used in the reinforced panels.

### 2.5. Boards BSG Preparation

#### 2.5.1. Particleboards Based on BSG & SPC

Dried BSG was blended with BSPC dispersion in three different solids ratios (see Table 1), in an orbital paddle mixer (Silcook, Guangzhou, China) for 10 min at room temperature. The resulting mixture was dried in an oven at 70 ± 2 °C until reaching a moisture content of 40 wt%. The boards were obtained by hot pressing (E.M.S., Rosario, Argentina) at 140 °C for 25 min and a pressure of 2.1 ± 0.1 MPa. For this purpose, a steel mold (300 mm × 300 mm), with stops to reach a constant thickness (5 mm), was used. These conditions were selected based on our previous experience with soybean protein concentrate-based particleboards and were chosen to provide sufficient time for water removal during hot pressing and to promote the thermally induced structural development and crosslinking of the soybean protein adhesive [33,42,43]. The amount of BSG-BSPC mixture placed in the mold was calculated to achieve the target density of 1000 kg/m^3^. Three types of boards were prepared using different BSPC content (Table 1). The three BSPC contents were selected based on previous studies with SPC-based particleboards and preliminary trials with BSG [19,42,43], covering a practical range from the minimum adhesive content that provided adequate board integrity to a relatively high adhesive content. At least four boards were manufactured for each formulation.

#### 2.5.2. Rosin-Coated Particleboards

The obtained B-BSPC boards were coated with a 1:3 (CL:EtOH) alcoholic rosin (colophony) solution using a paintbrush. The amount of coating applied was controlled gravimetrically to achieve a solution application rate of 22 g/m^2^. The coated boards were then dried at room temperature. The resulting samples were designated B-BSPC-CL.

#### 2.5.3. Wood Veneer-Reinforced BSG Boards

**Figure 1:**
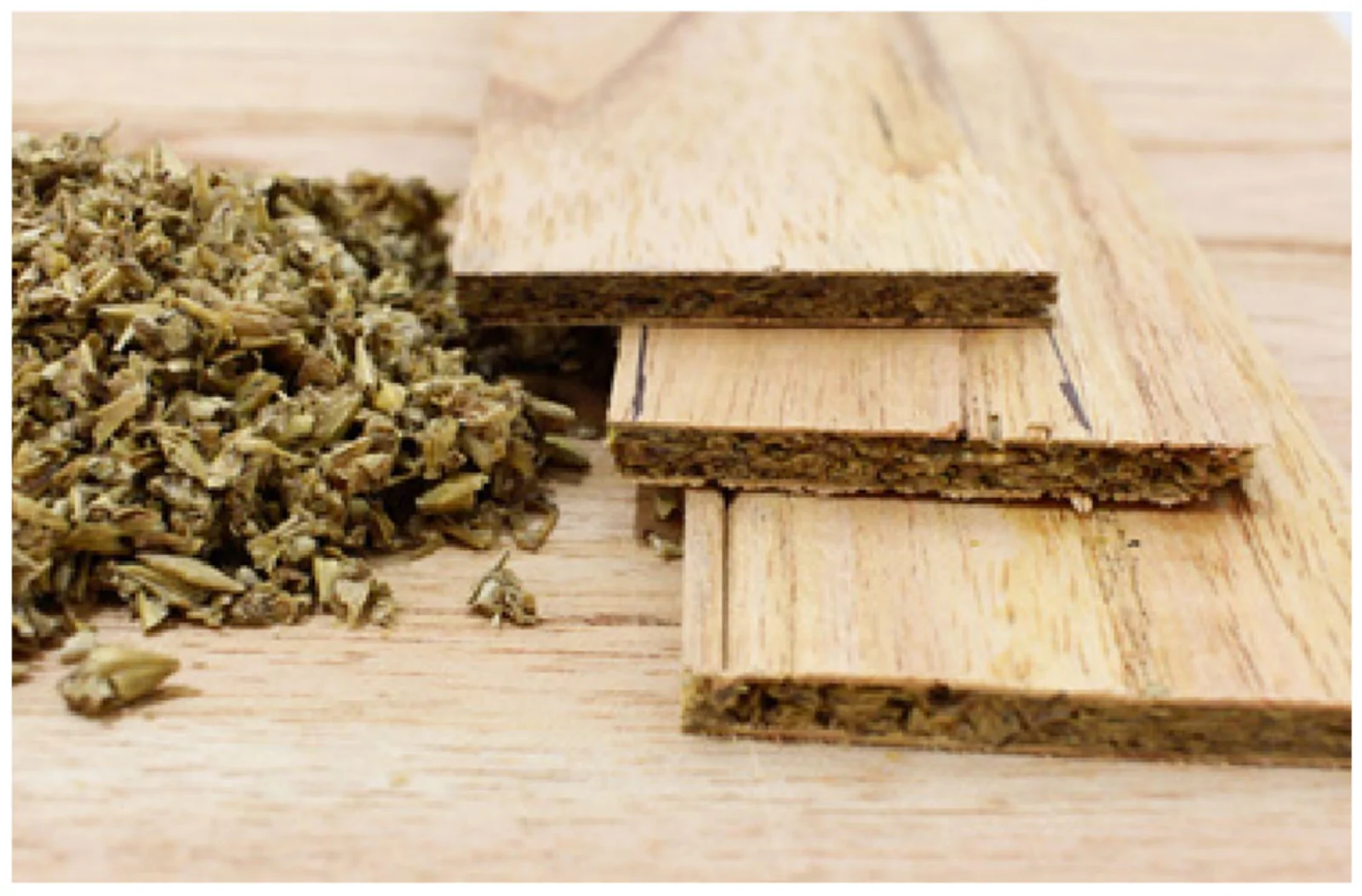
BSG wood-reinforced panels (B-BSPC-WR).

The boards were constructed as a three-layer assembly, consisting of a core formed by the B-BSPC mixture, prepared as described in Section 2.5.1, placed between two veneers manually coated with BSPC7 adhesive using a brush at an adhesive spread rate of 25 g/m^2^ (dry basis) (Figure 1). The mass of the mixture was adjusted to ensure that the density of the wood-reinforced panels was comparable to that of the B-BSPC panels. This approach ensured that any differences observed in the panel properties were attributable to the presence of wood layers rather than variations in density. The formulations were placed in a steel mold and hot-pressed under the same conditions described previously. Four boards, designated B-BSPC-WR, were produced to evaluate their performance.

### 2.6. Physical Properties of Panels

All board specimens, including rosin-coated and wood-reinforced boards, were conditioned in a climate chamber at 20 ± 2 °C and 65 ± 2% relative humidity until reaching equilibrium moisture content before evaluating their physical and mechanical properties.

#### 2.6.1. Density and Moisture Content

The moisture content (MC) and density (D) were determined in accordance with ASTM D1037-20 standard procedures [44]. Specimens measuring 50 mm × 50 mm × panel thickness were used to determine both properties. The thickness of the boards and the test specimens was determined using a digital caliper (Asimeto, Weißbach, Germany) with a resolution of 0.01 mm.

#### 2.6.2. Water Absorption and Thickness Swelling

Samples with dimensions of 50 mm × 50 mm × panel thickness were immersed in distilled water at room temperature to assess the water absorption (WA) and thickness swelling (TS) of the boards. The thickness and weight were measured at 2 h and 24 h after soaking, in accordance with ASTM D1037-20 or EN317-94 standard procedures.

#### 2.6.3. Water Diffusivity and Absorption

This analysis was adapted from Chalapud et al. (2023) [19]. Samples previously dried in a vacuum oven at 60 °C for 24 h, of 50 mm × 25 mm × panel thickness size, were evaluated in a constant humidity chamber at two different conditions of 50 and 80% RH at 20 °C. Samples were weighed systematically until the difference between two successive measurements was less than 0.001 g (m_i_). The equilibrated samples were dried at 105 °C and weighed (m_s_) in order to calculate ΔM (%), Equation (1), which was plotted as a function of the square root of time to obtain the slope (ΔM %/t^1/2^).

(1) $$\Delta M(\%)=\left(\frac{m_{i}-m_{s}\;}{m_{s}\;}\right)\;*\;100$$

The apparent diffusion coefficient (Da) was calculated with Equation (2), following Fick’s law:

(2) $$D_{a\;}=\;\pi \;*\;{\left(\frac{h}{4{\;M}_{m}}\right)}^{2}*{\left(\frac{{dM}_{t}}{d\sqrt{t}}\right)}^{2}\;$$

where h is the thickness of the sample, M_m_ is the moisture level at the saturation point, and dM_t_/dt^1/2^ is the slope obtained from the linear region. The real diffusion coefficient (D) was calculated using Equation (3):

(3) $$D\;=\;\frac{D_{a}}{\left[1\;+\;\frac{h}{L}\;+\;\frac{h}{W}\;\right]}$$

where L and W are the length and the width of the sample, respectively. The samples B-BSPC, B-BSPC-CL and B-BSPC-WR were evaluated in quintuplicate for each humidity percentage.

### 2.7. Evaluation of Mechanical Properties

All the produced boards were tested following the ASTM D1037-20 standard procedures. Three-point bending and tensile strength perpendicular to the panel surface (internal bond, IB) tests were performed using an Instron 4467 universal testing machine (Buckinghamshire, UK). A load cell of 10 kN with an accuracy of 0.2% was used for mechanical tests. Flexural properties, such as modulus of rupture (MOR) and modulus of elasticity (MOE), were determined from rectangular strips measuring 50 mm × 200 mm × thickness mm, employing a crosshead speed of 2.9 mm/min and a span of 130 mm. IB was estimated using square samples of 50 mm × 50 mm × thickness mm with a crosshead speed of 1.33 mm/min.

### 2.8. X-Ray Micro-Computed Tomography

To evaluate the internal structure of the particleboards, cross-sections of the samples were analyzed by X-ray microtomography (micro-CT) using a Bruker SKYSCAN 1273 (Bruker micro-CT, Karlsruhe, Germany). Tomographic projections were acquired at 57 kV and 250 µA over 180° with a 0.2° rotation step, using a 15 µm pixel size, four-frame averaging, and a 0.5 mm Al filter. The projections were reconstructed and rendered using CTvox software (version 3.3.0.r1412, 64-bit), allowing visualization of the internal structure of the selected board sections. The analysis was performed on sections with a thickness of 0.1 mm. This technique enables the identification of local density variations associated with the structural heterogeneity of the particleboard. The micro-CT analysis was used for qualitative comparison of the internal structure of the boards rather than for quantitative determination of porosity or absolute density.

### 2.9. Digital Image Correlation (DIC)

To investigate the deformation and failure behavior of BSG and wood-reinforced BSG boards (B-BSPC and B-BSPC-WR) under bending loads, a two-dimensional Digital Image Correlation (DIC) system (Correlated Solutions) was employed during flexural testing. The technique enabled full-field displacement and strain measurements throughout the loading process. Before testing, the edge of each specimen was painted with a uniform matte white background. A random black speckle pattern was then applied to the surface to create a high-contrast texture suitable for displacement and strain measurements. Images were acquired at a rate of 10 frames per second throughout the flexural test and recording was continued until complete specimen failure.

The experiments followed the evolution of the equivalent Green–Lagrange Von Mises strain fields of samples during the three-point bending tests. The equivalent Green–Lagrange Von Mises strain (E_vm_) is defined as [45]:

(4) $$E_{vm}=\sqrt{\frac{1}{2}\left({\left(E_{1}-E_{2}\right)}^{2}+{\left(E_{2}-E_{3}\right)}^{2}+{\left(E_{3}-E_{1}\right)}^{2}\right)}$$

Assuming plain strain conditions (E_3_ = 0), the expression reduces to:

(5) $$E_{vm}=\sqrt{E_{1}^{2}+E_{2}^{2}+E_{1}E_{2}}.$$

To objectively analyze the failure modes of the laminates, a surface kinematic triaxiality factor is introduced. This indicator is defined as the ratio of the first invariant of the strain tensor to the von Mises equivalent strain. Considering a plane strain condition (E_3_ ≈ 0) governed by the test kinematics, the expression reduces to:

(6) $$tr\frac{\left(E\right)}{E_{vm}}=\frac{E_{1}+E_{2}}{E_{vm}}$$

Physically, this factor accounts for the competition between local areal change and shape distortion. Values approaching zero indicate a deformation mode dominated by pure in-plane shear where material layers slide against each other with negligible local expansion or contraction. Conversely, positive or negative values reveal states dominated by localized tension or compression, respectively, allowing for a clear phenomenological identification of the fracture mechanisms prior to macroscopic collapse.

### 2.10. Statistical Analysis

Experimental data were statistically analyzed using the one-way analysis of variance (ANOVA, α = 0.05) along with Tukey’s tests at a 95% confidence interval.

## 3. Results and Discussion

### 3.1. Wood Characterization

#### Density, Moisture Content and Mechanical Properties

The density of the wood veneers was 625 ± 37 kg/m^3^, and their moisture content was 8.2 ± 0.3%. The tensile strength (TSt) and tensile modulus (TE) of the wood veneers were determined in the longitudinal (parallel to the fiber direction) and transverse (perpendicular to the fiber direction) directions (Table 2). The measured values were similar to those reported for the same species by other authors [46]. It is expected that reinforcing the panels with a wood veneer oriented in the longitudinal direction will significantly enhance their bending properties in that direction.

### 3.2. Particleboards Physical Characterization

#### 3.2.1. Density and Moisture Content

The mean density of the obtained panels was approximately 1000 kg/m^3^ (Table 3), with no statistically significant differences among the panels (*p* > 0.05). This value corresponds to the target global density defined for this study. According to the ANSI A208.1 standard, panels within this density range are classified as high-density panels. Similarly, no statistically significant differences in thickness were observed among the different panel types (*p* > 0.05), which can be attributed to the manufacturing method employed (Table 3). The MC values of the particleboards are shown in Table 3. No significant differences were observed among A-BSPC, B-BSPC, and C-BSPC (*p* > 0.05), indicating that the BSPC content did not significantly affect the moisture content of the BSG-based panels. In contrast, B-BSPC-WR and B-BSPC-CL, both containing the same BSPC content as B-BSPC, showed significantly different MC values (*p* < 0.05). These slight differences may be attributed to the incorporation of the external wood veneer or rosin, which may influence sorption and diffusion [47,48].

#### 3.2.2. Water Absorption and Thickness Swelling

Table 4 summarizes the water absorption (WA) and thickness swelling (TS) values measured after 2 and 24 h of water immersion. Among the unmodified boards, A-BSPC and B-BSPC showed similar behavior, whereas C-BSPC exhibited significantly lower WA and TS at both immersion times (*p* < 0.05). Thus, only the highest BSPC content produced a clear improvement in water resistance and dimensional stability. This result can be attributed to the higher adhesive-to-BSG ratio, which likely promoted more effective particle bonding and reduced the penetration of water into the board structure. A similar effect was reported by Rosenfeld et al. (2022) for particleboards bonded with a fructose-based adhesive, in which increasing the adhesive content reduced both WA and TS [49,50,51].

The rosin coating further improved the performance of the boards. Compared with B-BSPC, B-BSPC-CL exhibited significantly lower WA and TS after both 2 and 24 h of immersion (*p* < 0.05). The reduction in WA was particularly pronounced after 2 h, suggesting that the coating initially acted as an effective barrier to water penetration. Its protective effect persisted after 24 h, although the differences became smaller as immersion progressed. This behavior is consistent with the hydrophobic nature of rosin. Su et al. (2021) similarly observed that rosin treatment reduced the permeability and surface wettability of bamboo culms compared with untreated material [36,37].

The wood veneer reinforcement produced a different response. B-BSPC-WR showed significantly lower WA than B-BSPC after both immersion periods (*p* < 0.05), demonstrating that the veneer restricted water uptake. However, it did not significantly reduce TS (*p* > 0.05). In fact, despite the marked decrease in WA, the TS values of B-BSPC-WR remained statistically similar to those of B-BSPC. This indicates that limiting the amount of absorbed water was not sufficient to control thickness expansion. The veneer may act as an external barrier to water penetration, whereas TS remains mainly governed by the hygroscopic response and swelling of the compressed BSG-based core.

Overall, increasing the BSPC content and incorporating either a rosin coating or wood veneer reduced water absorption, although their effects on thickness swelling differed. The rosin coating improved both properties, whereas the wood veneer reduced only WA. The 2 and 24 h immersion times correspond to the standardized procedure used in ASTM D1037-20 and EN 317-94 and are intended to characterize water absorption and thickness swelling under the specified test conditions; they do not represent a long-term durability assessment. Nevertheless, the relatively high WA and TS values obtained after 24 h of immersion indicate that the boards are more suitable for dry or protected indoor environments than for applications involving direct water exposure. This limitation is characteristic of particleboards bonded with hydrophilic protein-based adhesives [52]. Although increasing the soy protein content can improve water resistance, it generally does not completely overcome this drawback [53]. Accordingly, Leiva et al. (2007) and Ciannamea et al. (2010) proposed dry indoor applications for rice husk particleboards bonded with soybean protein concentrate [42,43].

For applications requiring greater resistance to moisture or liquid water, an additional protective treatment may therefore be necessary. In this regard, Chalapud et al. (2023) reported that a polyurethane coating substantially improved the water resistance of rice husk–SPC particleboards [19].

#### 3.2.3. Moisture Absorption and Diffusivity

**Figure 2:**
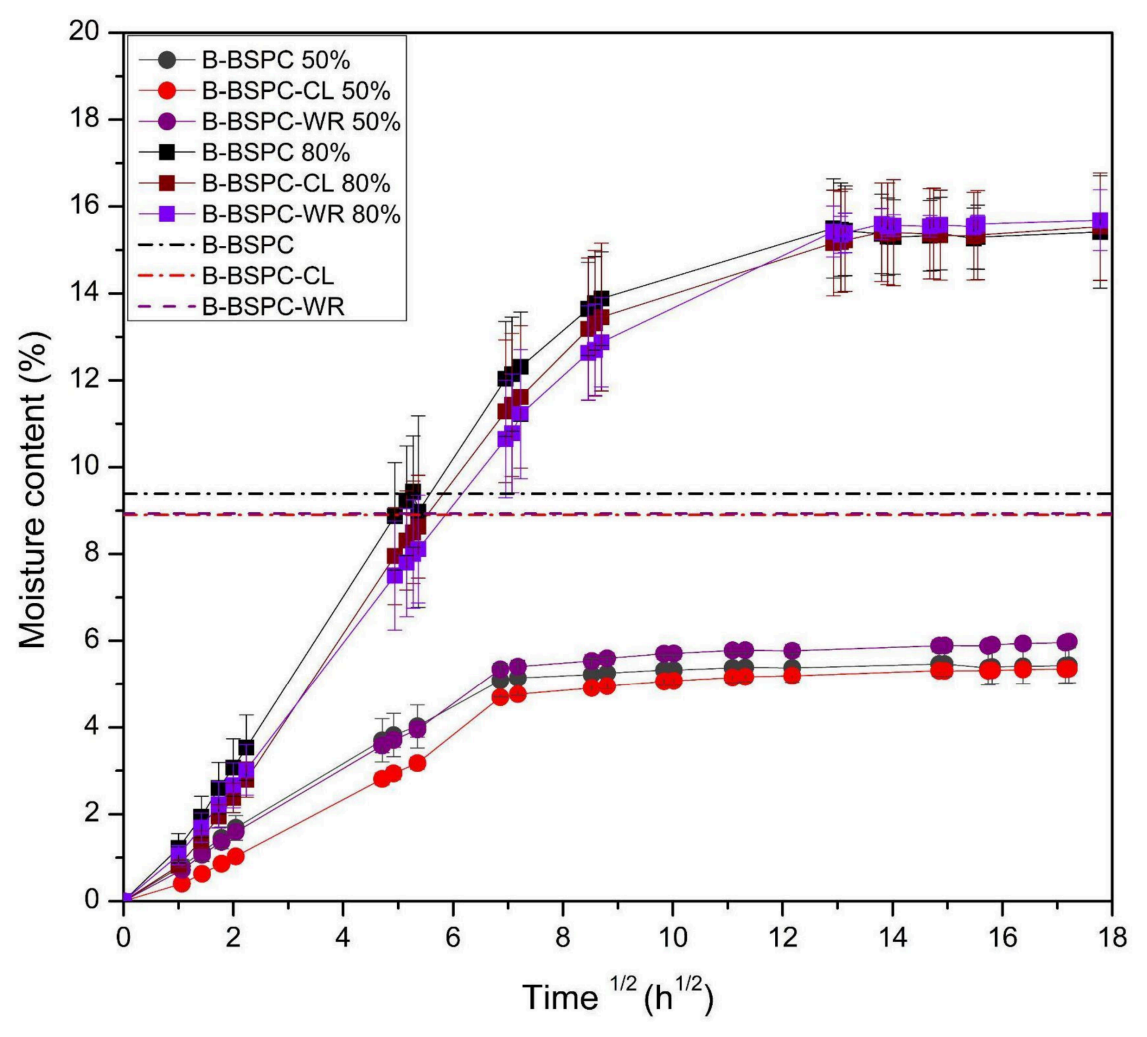
Moisture absorption curves of boards B-BSPC, B-BSPC-CL and B-BSPC-WR at different relative humidity levels, 50 and 80%. Dashed lines indicate the equilibrium moisture content of the corresponding samples at 65% RH, included as a reference.

The moisture sorption behavior of BSG-based particleboards is relevant for assessing their performance in humid environments. The evaluation of water diffusion and equilibrium moisture at two relative humidities (RH, 50 and 80%) is presented in Figure 2 as a function of the square root of time (h^1/2^). For these curves, the equilibrium moisture content (EMC), equilibrium moisture time (EMT), apparent diffusion coefficient (Da) and real diffusion coefficient (D) were estimated, and the results are summarized in Table 5. EMC was determined for each sample at the time when the moisture content reached a constant value.

The diffusion coefficients (Da and D) were obtained from the slope of the linear region of the curves shown in Figure 2; values are shown in Table 5. At 50% RH, both modified boards exhibited significantly lower D (*p* < 0.05) than the reference board, indicating a slower rate of moisture transport within the board structure (Table 5). The B-BSPC-WR board exhibited significantly lower diffusion coefficients than B-BSPC and B-BSPC-CL, at 80% RH, suggesting that the presence of the wood layers further limited moisture transport under high humidity conditions.

As expected, increasing RH from 50 to 80% substantially increased the equilibrium moisture content of all boards. However, the EMC values were not significantly affected by the modifications at either RH (*p* > 0.05), indicating that the treatments primarily influenced the moisture transport kinetics rather than the final equilibrium moisture content.

The time required to reach equilibrium moisture was also longer for the modified boards (B-BSPC-CL and B-BSPC-WR) than for the reference board B-BSPC. Chalapud et al. (2020) reported similar behavior for tung oil-impregnated rice husk-based particleboards, where an increase in the time to reach equilibrium moisture content and a reduction in the diffusion coefficients were attributed to the penetration of hydrophobic tung oil into the voids of the particleboard, which imparted hydrophobic characteristics and limited the diffusion of water molecules into the board matrix [33]. In the present study, the rosin coating and the incorporation of wood layers reduce the accessibility of hydrophilic sites and partially block void spaces within the particle structure, thereby hindering the diffusion of water molecules through the board matrix. These results indicate that superficial rosin coating provides a simple approach to reduce moisture transport under humid atmospheres, without requiring the incorporation of additional wood layers.

#### 3.2.4. Mechanical Properties of Particleboards

Table 6 presents the mean MOR, MOE, and IB values of the manufactured particleboards, together with the requirements established by American and European standards for indoor applications under dry service conditions. All three mechanical properties increased progressively from A-BSPC to C-BSPC, with significant differences among the formulations (*p* < 0.05), demonstrating the beneficial effect of increasing the BSPC content. This improvement is closely related to the higher adhesive-to-particle ratio. Because board density was maintained constant, increasing the BSPC content necessarily reduced the proportion of BSG particles and increased the amount of adhesive available to bond them. During hot pressing, this greater adhesive availability likely promoted more complete wetting and coverage of the particle surfaces, penetration into surface pores and exposed cell lumina, and the formation of more continuous bond lines between adjacent particles. These processes increase the effective contact area and favor mechanical interlocking within the porous lignocellulosic structure. They also facilitate intermolecular interactions, particularly hydrogen bonding between the hydroxyl groups of the lignocellulosic substrate and the polar functional groups of soy proteins [18,25,33,47]. Together, these effects strengthen interparticle bonding and improve stress transfer throughout the board structure, resulting in higher MOR, MOE, and IB values. Similar trends have been reported for lignocellulosic particleboards manufactured with increasing contents of soy protein isolate, wheat gluten-based adhesives, or synthetic resins [49,50,51,52].

Increasing the BSPC content from the A-BSPC to the C-BSPC formulation resulted in increases of 178, 156, and 226% in MOR, MOE, and IB, respectively. Despite these substantial improvements, the flexural properties remained below the minimum requirements established by the European and American standards. Therefore, although increasing the BSPC content enhanced particle bonding and stress transfer, it was insufficient to overcome the limited load-bearing capacity of the BSG particles. This limitation may be associated with the relatively low cellulose content of BSG compared with wood and other lignocellulosic residues, such as rice husk, which reduces its ability to reinforce the board structure. These results indicate that an additional reinforcement strategy is required to achieve the flexural performance demanded by the standards.

Post-pressing rosin treatment also improved the mechanical performance of the boards. B-BSPC-CL exhibited significantly higher MOR, MOE, and IB values than untreated B-BSPC (*p* < 0.05). Because rosin was incorporated after board manufacture, it did not participate in the formation of the initial adhesive bonds during hot pressing. Instead, its penetration into the board and subsequent deposition within pores and at particle–adhesive interfaces may have helped consolidate the internal structure. Moreover, by limiting moisture uptake, rosin may have reduced plasticization of the soy protein matrix and contributed to preserving interfacial integrity, thereby improving stress transfer. Nevertheless, the individual contributions of these mechanisms cannot be distinguished from the present results. No direct evidence of specific chemical interactions between rosin, SPC, and BSG was obtained in this study. Comparable improvements in mechanical properties, water resistance, and dimensional stability have been reported for other rosin-treated lignocellulosic materials [36,37,47]. Similarly, Chalapud et al. (2020) found that post-manufacturing tung oil impregnation improved both the physical and mechanical performance of rice husk particleboards bonded with soybean protein concentrate [33].

The incorporation of external wood veneers substantially improved the flexural performance of the BSG-based particleboards. The veneers were positioned on the outer surfaces of the boards, with their fibers preferentially oriented along the main bending direction. Accordingly, B-BSPC-WR exhibited significantly higher MOR and MOE values than unreinforced B-BSPC (*p* < 0.05) and met the flexural requirements established for the H1 and P2 classifications in ANSI A208.1 and EN 312, respectively. These results indicate that wood veneer reinforcement effectively compensated for the relatively low stiffness and strength of the BSG-based core.

This improvement can be explained by the mechanics of layered materials under bending. During flexural loading, the regions below and above the neutral axis are subjected predominantly to tensile and compressive stresses, respectively, with the maximum stresses occurring at the outermost surfaces. Because the wood veneers were located in these highly stressed regions and had a substantially higher elastic modulus and strength than the particleboard core, they carried a large proportion of the tensile and compressive loads. Moreover, their distance from the neutral axis increased their contribution to the flexural rigidity of the board. Consequently, the veneers enhanced both the resistance to bending deformation, reflected in the higher MOE, and the maximum load-bearing capacity, reflected in the higher MOR. The pronounced increase in MOR and MOE, together with the deformation patterns observed by DIC (discussed further below), is consistent with effective load transfer between the wood veneers and the BSG–SPC core.

The IB strength was not evaluated for B-BSPC-WR boards because the standard IB test is intended to assess the cohesive strength under tensile loading perpendicular to the panel plane of a conventional particleboard core. Since the wood veneer reinforcement was confined to the outer surfaces and did not modify the composition or structure of the BSG-SPC core, evaluating IB would not provide additional information on the effect of the veneer reinforcement on the core itself.

#### 3.2.5. X-Ray Microtomography

**Figure 3:**
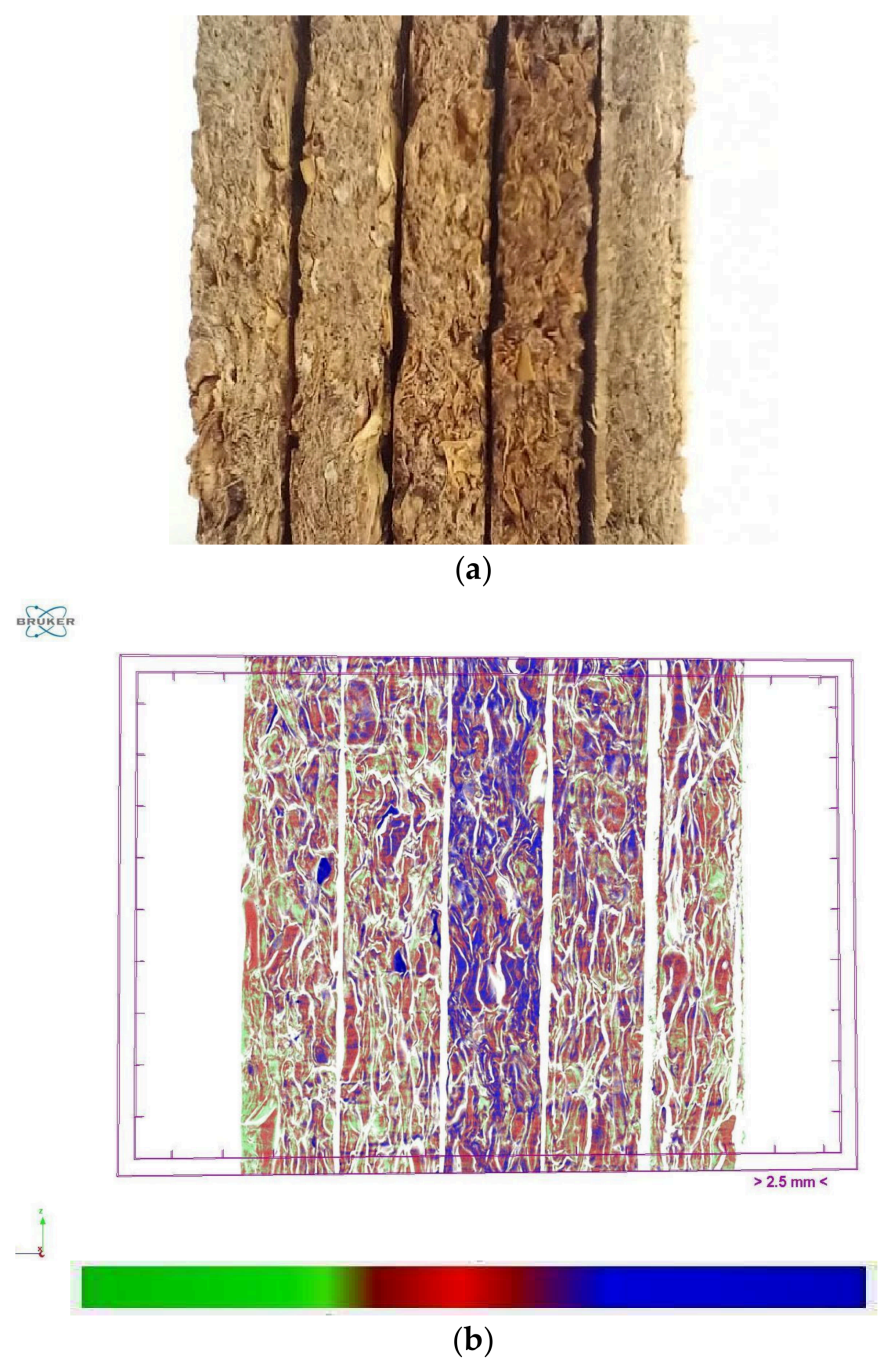
(**a**) Cross-section of particleboards from left to right: A-BSPC, B-BSPC, C-BSPC, B-BSPC-CL and B-BSPC-WR, (**b**) micro-CT reconstructed image of particleboards and colorbar showing density scale for micro-images; density increases slightly from green to blue. The white regions correspond to pores or areas of very low local density.

The micro-CT images of the board cross-sections (Figure 3) revealed differences in the spatial distribution of local density among the samples. From A-BSPC to C-BSPC, a greater presence of blue regions was observed, indicating areas of higher local density as the adhesive content increased. However, these differences should not be interpreted as an increase in the global apparent density of the boards. All formulations exhibited similar apparent densities, as determined from their mass-to-volume ratios, with no statistically significant differences among them. Therefore, the higher-density regions observed in the micro-CT images are compensated by pores or areas of lower local density elsewhere within each board. Thus, micro-CT provides information on the internal distribution of density that cannot be obtained from global apparent-density measurements.

Compared with B-BSPC, the B-BSPC-CL cross-section displayed additional regions of higher local density, particularly near the board surfaces. These regions are consistent with the penetration and deposition of the rosin applied after board manufacture. Nevertheless, because the global apparent density of the boards remained unchanged, these local increases in density are compensated by pores or lower-density regions within the board cross-section.

The B-BSPC-WR board exhibited a BSG–BSPC core with a local-density distribution generally similar to that of B-BSPC. The external wood veneers are not clearly visible in the image because their lower local density causes them to appear white and blend with the background. Consequently, the B-BSPC-WR board appears thinner than the other boards in the micro-CT image, although its actual thickness includes the two external veneers. This low contrast is consistent with the density of the wood used for the veneers (625 kg/m^3^), which was lower than the apparent density of the BSG-based core (approximately 1000 kg/m^3^).

#### 3.2.6. DIC

**Figure 4:**
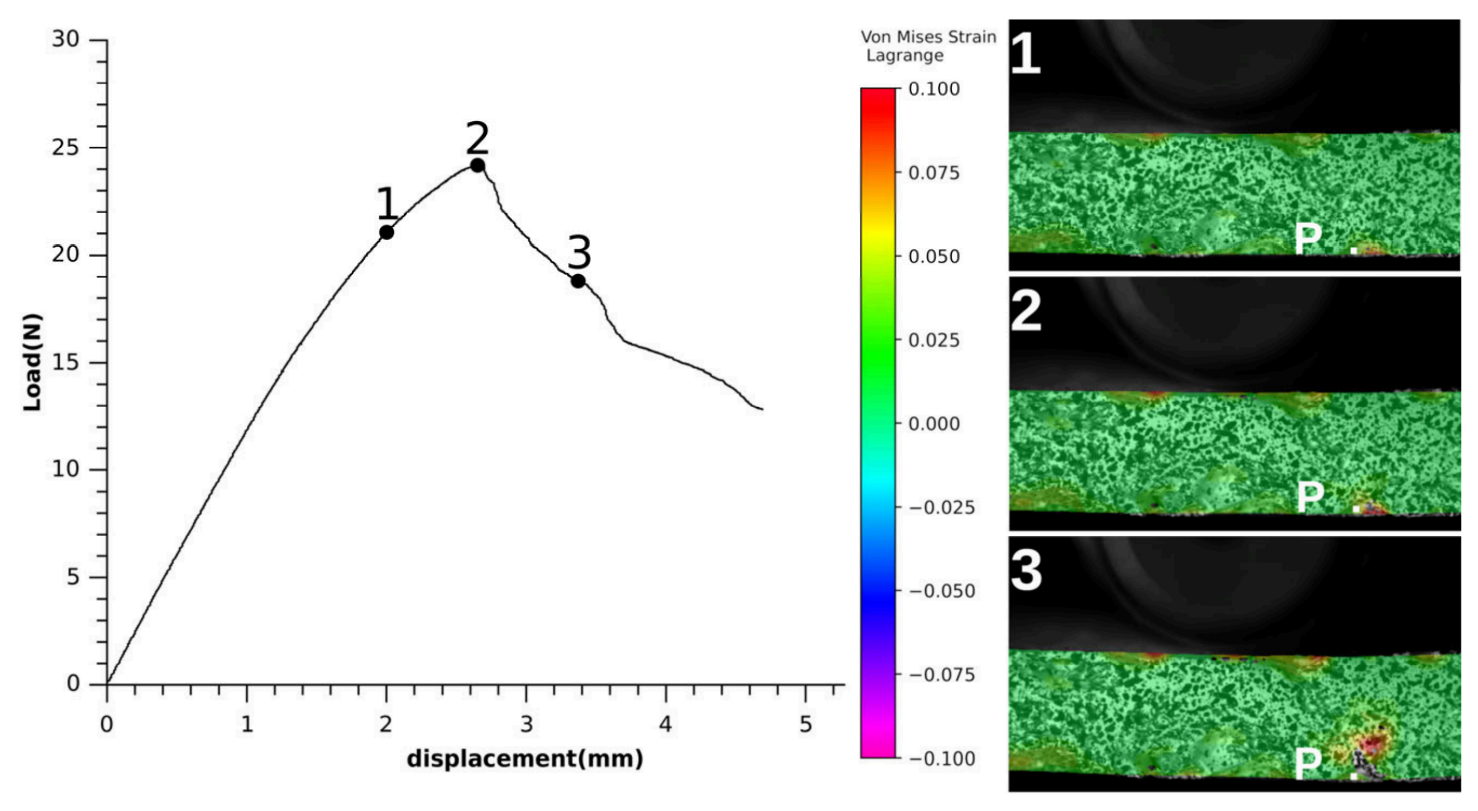
Evolution of the equivalent Green–Lagrange Von Mises strain fields of B-BSPC boards during the three-point bending tests. The letter P marks the specific region of interest where failure develops.

**Figure 5:**
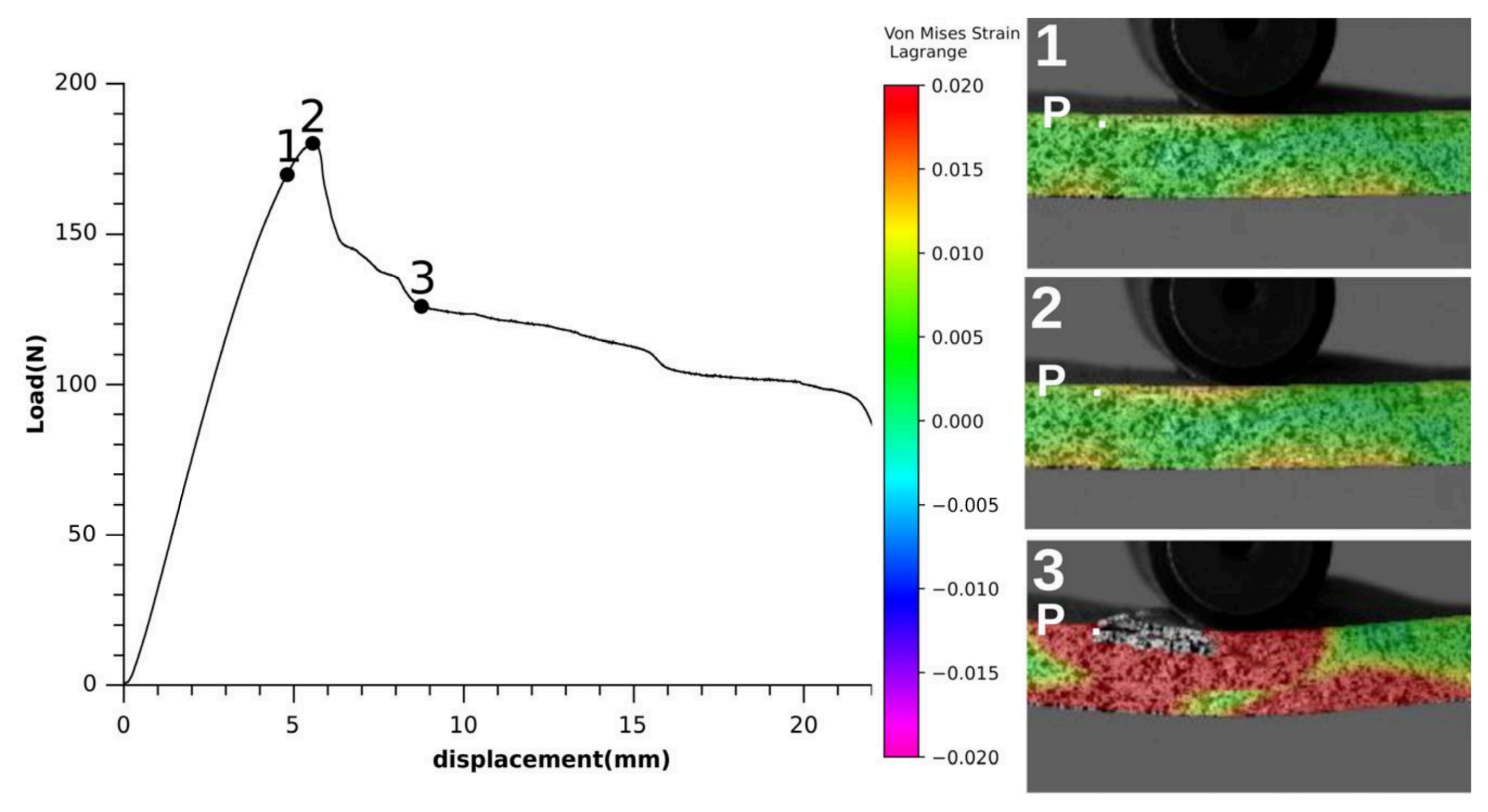
Evolution of the equivalent Green–Lagrange Von Mises strain fields of B-BSPC-WR boards during the three-point bending tests. The letter P marks the specific region of interest where failure develops.

Figure 4 and Figure 5 show the evolution of the equivalent Green–Lagrange Von Mises strain fields of B-BSPC and B-BSPC-WR boards during the three-point bending tests.

For both materials, the DIC analysis revealed progressive strain localization prior to failure. At points 1 and 2, deformation remained relatively distributed over the specimen, although regions of increasing equivalent strain became visible near the loading area. After reaching the maximum load, deformation became increasingly localized, leading to the formation of concentrated strain regions associated with damage development and failure progression. Although both materials exhibited strain localization in the equivalent Von Mises strain field prior to failure, further analysis of the individual strain components suggests different deformation mechanisms, consistent with the failure modes observed by naked-eye inspection.

**Figure 6:**
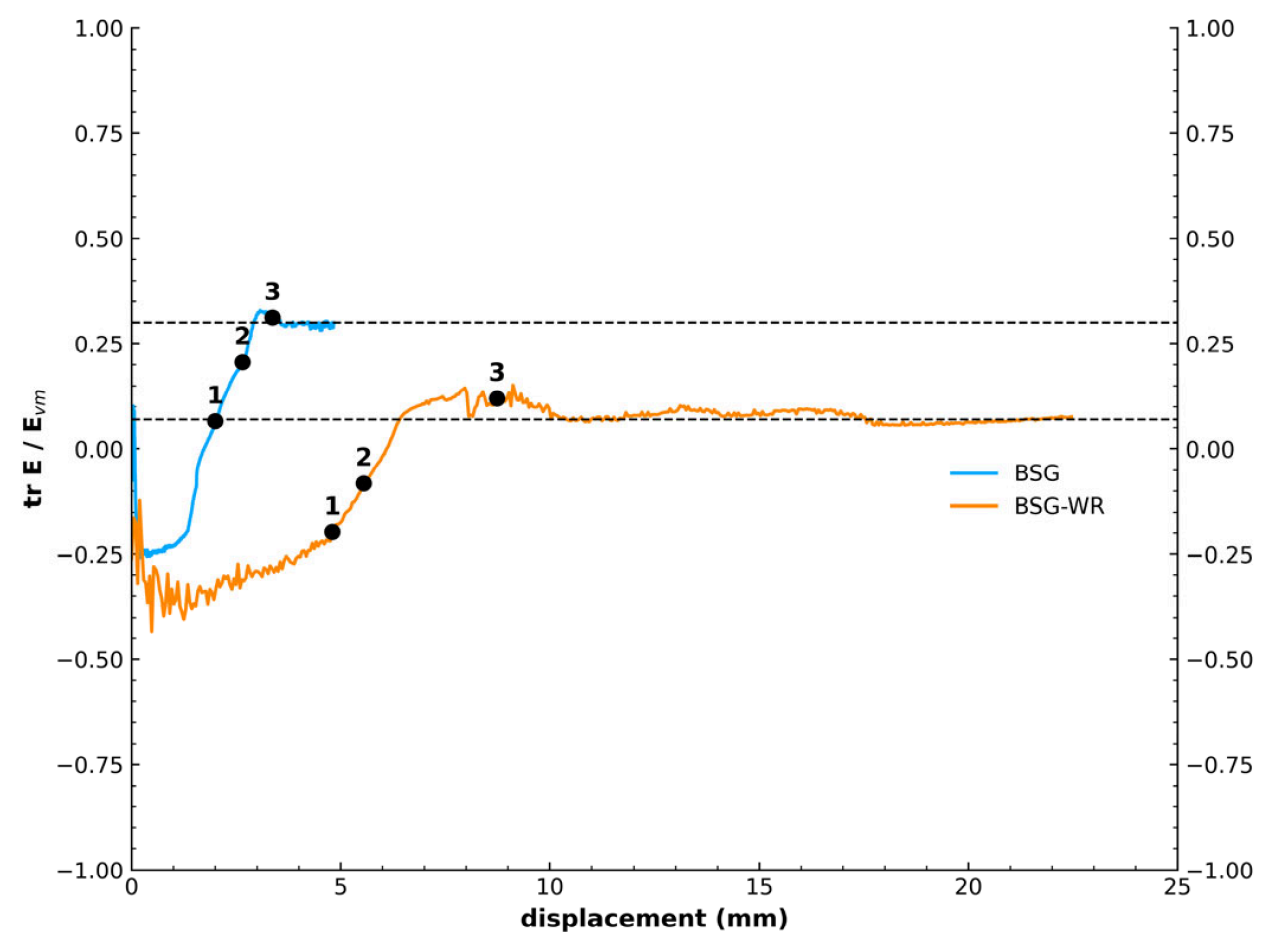
Evolution of the kinematic triaxiality factor, tr(E)/Evm, as a function of displacement for both B-BSPC and B-BSPC-WR boards calculated at the failure zone P (identified in Figure 4 and Figure 5). The horizontal dashed lines indicate the steady-state reference levels to which the factors tend.

Figure 6 shows the evolution of the kinematic triaxiality factor, tr(E)/E_vm_, as a function of displacement for both B-BSPC and B-BSPC-WR boards. For reference, points of interest 1, 2, and 3 corresponding to Figure 4 and Figure 5 are indicated. In both materials, the curves plateau after the onset of failure (point 2), revealing distinct material behaviors. For the unreinforced BSG, the indicator stabilizes at a positive value (approx. 0.3). Although this purely kinematic parameter cannot be directly equated to the classic stress triaxiality values, its positive magnitude indicates a failure mechanism with a significant tensile-driven areal expansion. Conversely, for the B-BSPC-WR boards, the factor drops and tends to zero. This confirms that the macroscopic failure in the reinforced boards is heavily dominated by in-plane shear, where material layers undergo relative sliding with negligible local areal expansion. Consequently, the ability to objectively capture these distinct behaviors lays the groundwork for designing tailored damage indicators specific to each material configuration in future studies.

## 4. Conclusions

Increasing the BSPC content simultaneously improved the water resistance and mechanical performance of the BSG-based particleboards. Because all formulations had similar global apparent densities, these improvements were attributed to the higher adhesive-to-particle ratio rather than to greater board densification.

Post-pressing rosin treatment further reduced WA and TS while increasing MOR, MOE, and IB. Micro-CT analysis showed some regions with higher local density near the board surfaces, which may be consistent with the penetration and deposition of rosin within the porous structure.

External wood veneers provided the most effective improvement in flexural performance. Their high stiffness and location on the outer surfaces of the boards significantly increased MOR and MOE, allowing B-BSPC-WR to meet the minimum flexural requirements for the H1 and P2 classifications established by ANSI A208.1 and EN 312, respectively, for indoor applications under dry or protected service conditions. DIC analysis further showed that the veneers modified the failure mechanism, shifting the response from tensile-driven crack opening in B-BSPC to predominantly deviatory deformation, consistent with shear and interlayer sliding in B-BSPC-WR.

The combined use of brewer’s spent grain, a bio-based and formaldehyde-free soybean protein concentrate adhesive, and wood veneer reinforcement represents a promising approach to producing more sustainable particleboards with enhanced flexural performance. This strategy valorizes an agro-industrial residue, reduces wood consumption, and avoids the potentially harmful emissions associated with conventional formaldehyde-based adhesives.

## Figures and Tables

**Table 1:** Particleboard identification and adhesive specifications.

Sample Name	Adhesive	Adhesive Content (wt% Dry Basis)
A-BSPC	BSPC	9.51 ± 0.08a
B-BSPC	BSPC	17.43 ± 0.04b
C-BSPC	BSPC	24.72 ± 0.76c

Mean values in the same column followed by different letters are significantly different (*p* < 0.05) by Tukey’s Test.

**Table 2:** Tensile strength (TSt) and tensile modulus (TE) of wood veneers measured in longitudinal (L) and transverse (T) orientations.

Fiber Orientation	TSt (MPa)	TE (GPa)
L	63.3 ± 3.1	8.5 ± 0.5
T	1.6 ± 0.3	1.1 ± 0.2

**Table 3:** Density and MC of prepared particleboards.

Sample	Density (kg/m^3^)	Thickness (mm)	MC (%)
A-BSPC	986 ± 25a	4.83 ± 0.05a	9.34 ± 0.23a
B-BSPC	988 ± 38a	4.82 ± 0.04a	9.39 ± 0.17a
C-BSPC	1015 ± 66a	4.75 ± 0.10a	9.45 ± 0.21a
B-BSPC-CL	991 ± 29a	4.77 ± 0.11a	8.90 ± 0.03b
B-BSPC-WR	1010 ± 80a	4.88 ± 0.11a	8.93 ± 0.06b

Mean values in the same column followed by different letters are significantly different (*p* < 0.05) by Tukey’s Test.

**Table 4:** WA and TS, at 2 h and 24 h of water immersion, of prepared particleboards.

Sample	WA/2 h	WA/24 h	TS/2 h	TS/24 h
A-BSPC	120.8 ± 18.6a	135.9 ± 18.8a	51.5 ± 10.4a	82.7 ± 17.6a
B-BSPC	114.1 ± 9.9a	134.9 ± 9.5a	54.9 ± 9.8a	80.8 ± 7.8a
C-BSPC	75.9 ± 17.4b	106.8 ± 15.1b	41.1 ± 5.6b	57.2 ± 6.2b
B-BSPC-CL	62.7 ± 11.4b	105.7 ± 9.6b	37.9 ± 5.1b	68.7 ± 8.5b
B-BSPC-WR	67.6 ± 6.7b	99.4 ± 9.5b	64.3 ± 9.5a	75.7 ± 11.5a

Mean values in the same column followed by different letters are significantly different (*p* < 0.05) by the Tukey Test.

**Table 5:** Equilibrium moisture content (EMC), equilibrium moisture time (EMT), apparent diffusion coefficient (Da) and real diffusion coefficient (D) of particleboard samples at two relative humidity levels (50 and 80%).

Sample	RH (%)	EMC (%)	EMT (h^1/2^)	Da × 10^5^ (mm^2^/s)	D × 10^5^ (mm^2^/s)
B-BSPC	50	5.6 ± 0.3a	11.1	2.38 ± 0.22a	1.85 ± 0.29a
B-BSPC-CL	50	5.4 ± 0.1a	14.8	1.98 ± 0.06b	1.53 ± 0.05b
B-BSPC-WR	50	5.7 ± 0.3a	14.8	2.04 ± 0.08a,b	1.54 ± 0.05b
B-BSPC	80	15.4 ± 1.3b	12.9	1.68 ± 0.08c	1.31 ± 0.06c
B-BSPC-CL	80	15.5 ± 1.2b	13.9	1.62 ± 0.17c	1.25 ± 0.13c
B-BSPC-WR	80	15.6 ± 0.7b	17.8	1.17 ± 0.06d	0.88 ± 0.04d

Mean values in the same column at equal RH followed by different letters are significantly different (*p* < 0.05) according to Tukey’s test.

**Table 6:** Mechanical properties of prepared particleboards.

Sample	MOR (MPa)	MOE (GPa)	IB (MPa)
A-BSPC	1.95 ± 0.34a	0.65 ± 0.14a	0.15 ± 0.03a
B-BSPC	2.83 ± 0.54b	0.95 ± 0.17b	0.24 ± 0.04b
C-BSPC	5.33 ± 0.94c	1.67 ± 0.27c	0.49 ± 0.14c
B-BSPC-CL	4.21 ± 0.95c	1.52 ± 0.20c	0.33 ± 0.02d
B-BSPC-WR	24.1 ± 6.9d	3.52 ± 0.87d	-
H1(ANSI208)	16.5	2.4	0.9
P2 (EN312)	12.0	1.95	0.45

Mean values in the same column followed by different letters are significantly different (*p* < 0.05) according to Tukey’s test.

## Data Availability

The data presented in this study are available on request from the corresponding author.

## Abbreviations

The following abbreviations are used in this manuscript:

BSG	Brewer’s spent grain
SPC	Soybean Protein Concentrate
BSPC	Soybean Protein Concentrate-based adhesive
X-ray micro-CT	X-ray micro-computed tomography
DIC	Digital Image Correlation
MOE	Modulus of elasticity
MOR	Modulus of rupture
IB	Internal bond
WA	Water absorption
TS	Thickness swelling
RH	Relative humidity
MC	Moisture content
EMC	Equilibrium moisture content
EMT	Equilibrium moisture time
Da	Apparent diffusion coefficient
D	Real diffusion coefficient
TSt	Tensile strength
TE	Tensile modulus
L	Longitudinal
T	Transverse
